# Spatially Multiplexed Micro-Spectrophotometry in Bright Field Mode for Thin Film Characterization

**DOI:** 10.3390/s16060926

**Published:** 2016-06-21

**Authors:** Valerio Pini, Priscila M. Kosaka, Jose J. Ruz, Oscar Malvar, Mario Encinar, Javier Tamayo, Montserrat Calleja

**Affiliations:** IMM-Instituto de Microelectrónica de Madrid (CNM-CSIC), Isaac Newton 8, PTM, Tres Cantos, E-28760 Madrid, Spain; priscila@imm.cnm.csic.es (P.M.K.); jose.ruz@imm.cnm.csic.es (J.J.R.); oscar.malvar@csic.es (O.M.); mario.encinar@csic.es (M.E.); jtamayo@imm.cnm.csic.es (J.T.); mcalleja@imm.cnm.csic.es (M.C.)

**Keywords:** nanomechanics, microcantilever sensors, nanomechanical sensors, thin film, microspectrophotometry, resonators, mass sensing, surface stress

## Abstract

Thickness characterization of thin films is of primary importance in a variety of nanotechnology applications, either in the semiconductor industry, quality control in nanofabrication processes or engineering of nanoelectromechanical systems (NEMS) because small thickness variability can strongly compromise the device performance. Here, we present an alternative optical method in bright field mode called Spatially Multiplexed Micro-Spectrophotometry that allows rapid and non-destructive characterization of thin films over areas of mm^2^ and with 1 μm of lateral resolution. We demonstrate an accuracy of 0.1% in the thickness characterization through measurements performed on four microcantilevers that expand an area of 1.8 mm^2^ in one minute of analysis time. The measured thickness variation in the range of few tens of nm translates into a mechanical variability that produces an error of up to 2% in the response of the studied devices when they are used to measure surface stress variations.

## 1. Introduction

The knowledge of the thickness of a thin film is of primary importance in a large variety of application fields ranging from fabrication of integrated circuits in the semiconductor industry [1], engineering of optical coatings [2], optimization of thin-film batteries [3], photovoltaic cells [4] and the quality control of nanofabrication processes [5].

Optical techniques, such as imaging ellipsometry [6,7,8], white light interferometry [9,10,11,12,13,14,15,16] and micro-spectrophotometry [17,18,19,20,21,22] are widely used experimental methods for thin film thickness characterization. These are nondestructive methods, they do not require any previous sample preparation and can easily achieve spatial lateral resolution in the micrometer range. Imaging ellipsometry is a versatile technique for the non-destructive optical characterization of thin films [6,7,8]. However, in this technique, a motorized focusing system is needed because measurements need to be performed at varying observation angles [8]; this experimental requirement increases the measurement time and adds complexity to the entire equipment. White light interferometry has been implemented during the last decades for the simultaneous measurement of both surface topography and film thickness [9,10,11,12,13,14,15,16,23]. Despite its excellent vertical resolution, the complex mixture of overlapped interference signals make it challenging to decouple the film thickness and top surface topography from the measurements [10,11]. Micro-spectrophotometry is a well-known technique for the thickness characterization of a sample surface with high spatial resolution [19]. In these instruments, a white light beam is tightly focused onto the sample surface through a microscope objective and the reflected or transmitted light coming from the sample is collected and coupled to the entrance aperture of an optical spectrometer. One of the drawbacks of a standard micro-spectrophotometers is the fact that measurements can be performed only onto a specific and small area of few µm^2^, so the spatial thickness mapping on extended areas requires the movement of the sample with a control stage, which is usually tedious, costly and time-consuming [19,20]. For a fixed spatial resolution, the time measurement scales quadratically with the sample size to be detected, thus limiting the use of this technique as an efficient characterization and quality control tool in science and industry. 

In this work, we overcome the cited drawback of standard micro-spectrophotometry by using a novel optical technique, called Spatially Multiplexed Micro-Spectrophotometry (SMMS), which is able to perform fast thickness characterization of millimeter size samples with lateral spatial resolution of 1 μm. The SMMS technique represents a very general method for the characterization of thin films with low and moderate optical absorption. SMMS in bright field mode is presented here and applied to determine the thickness of an entire array of commercial silicon microcantilevers with a total measurement area of 3.6 mm^2^, and a lateral resolution of 1 µm and 1 nm vertical resolution, keeping measurement time down to 2 min. The present technique now makes it possible to observe small thickness inhomogeneities and structural defects over large devices in the mm range, thus representing a useful tool in nanotechnology for the assessment of the mechanical variability of a device due to the nanofabrication process.

## 2. Experimental Details

### 2.1. Spatially Multiplexed Micro-Spectrophotometry (SMMS) in Bright Field Mode 

**Figure 1:**
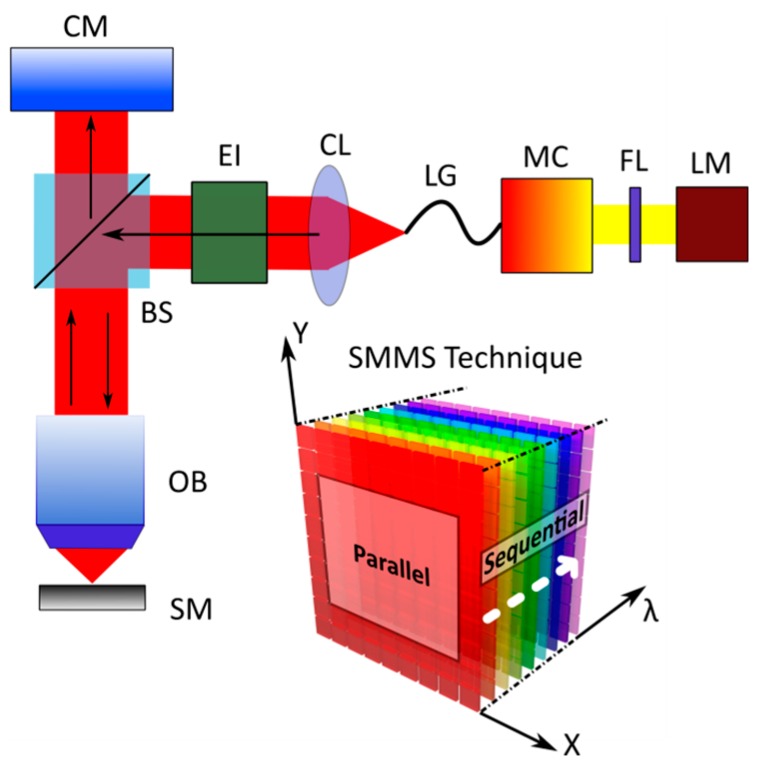
Schematic representation of the SMMS technique in reflection mode configuration. The right bottom inset is a schematic drawing of the SMMS working principle (spectral cube); the measurements are acquired in parallel for the spatial coordinates X and Y, and sequentially along the spectral coordinate λ.

The spectral characterization of the reflected light by a sample needs the acquisition of a tridimensional data set known in literature as a spectral cube; the first two coordinates *X* and *Y* of the spectral cube correspond to the sample surface while the third coordinate λ represents the light wavelength. In standard micro-spectrophotometers, the spectral analysis of a single point on the sample surface is acquired in a one-shot measurement, while the spatial mapping is performed by sequentially scanning point-by-point the sample surface. Micro-spectrophotometers are thus parallel in the spectral coordinate λ and sequential in the spatial coordinates *X* and *Y*. The SMMS technique described here performs one shot measurement of the reflected light coming from an extended sample area at each fixed wavelength, and sweeps sequentially over the desired range of wavelengths. Thus, as shown in the schematic drawing of Figure 1, the SMMS technique is parallel in the spatial coordinates (*X,Y*), whereas it is sequential in the spectral analysis (λ).

A practical realization of the SMMS instrument in reflection mode configuration is shown in Figure 1. The white light coming from a Xenon lamp (LM, PowerArc™, Optical Build Blocks, Edison, NJ, USA) is directed to a motorized monochromator (MC, OB-2000, Optical Build Blocks, Edison, NJ, USA) that disperses the light into its constituent wavelengths. A narrow band of the dispersed spectrum passing from the exit slit of the monochromator is directed to a collimating adapter (CL, LLG5A5-A, Thorlabs, Newton, NJ, USA) through a liquid light guide (LG, LLG0538-6, Thorlabs, Newton, NJ, USA). Thus, monochromatic and collimated light is coupled to a microscope epi-illuminator (EI, LV-UEPI, Nikon, Tokyo, Japan) and focused on the sample (SM) by means of a beam splitter (BS, Nikon) and a bright-field objective (OB, LU Plan Fluor, Nikon, Tokyo, Japan). A high resolution Peltier-cooled color charge-coupled device (CCD) camera (CM, DS-Ri1, Nikon, Tokyo, Japan) placed at the image plane of the experimental setup collects, for each illumination wavelength, λ, the reflected light coming from an extended sample surface. In order to avoid the overlapping of the second-order diffraction of light coming from the monochromator, a long-wave pass optical filter (FL, GG475, Microbeam, Barcelona, Spain) was placed along the illumination arm of the experimental setup. The use of an adjustable diaphragm placed inside the epi-illuminator allows tuning the numerical aperture of the light illumination from zero to a maximum angle defined by the numerical aperture of the objective used. The monochromator light wavelength is routinely calibrated by using a high quality band-pass filter (FL 05632.8-1, Thorlabs, Newton, NJ, USA, center wavelength λ = 632.8 ± 0.2 nm) that ensures a control of the light wavelength with an uncertainty of 0.2 nm. In order to eliminate the wavelength-dependence of the SMMS system response and the spatial inhomogeneity of the lamp illumination, raw data need to be normalized with the reflectivity spectra of a reference material; more technical details about raw data normalization can be found in Appendix A. The described SMMS instrument can also be employed as a standard optical microscope by making use of the zero-order diffraction of the monochromator grating. 

The SMMS technique is presented in this work in bright-field mode, but it can also be implemented in dark-field mode, as long as the appropriate optical components are used; more technical details about the SMMS technique in dark-field mode can be found elsewhere [24]. The measurement of large sample areas using the SMMS technique (typical range from several hundreds of µm^2^ up to few cm^2^, depending on the optical objective and CCD camera used) is much faster than standard micro-spectrophotometric techniques, as the use of control stages for the movement of the sample is no longer required. Moreover, the absence of an optical fiber along the detection arm, a common element in standard micro-spectrophotometers, ensures better robustness and easier maintenance of the SMMS instrument. In the following, we demonstrate the capability of the SMMS technique in bright field mode for thin film characterization.

### 2.2. Bright Field Spectral Analysis of Commercial Cantilevers

Standard microcantilevers are excellent candidates to test the capability of the SMMS technique for thin film thickness characterization. Cantilevers, broadly used both in atomic force microscopy and in cantilever sensing applications [25,26], are usually fabricated by standard microlithography technologies. Thickness inhomogeneity in the suspended structures can compromise the performance of the fabricated devices [27,28]. Moreover, the industrial quality control of nanofabrication processes requires of a high throughput technique because large volume inspections are needed and a high spatial resolution is indispensable as the size under inspection reduces with ever-increased package density.

**Figure 2:**
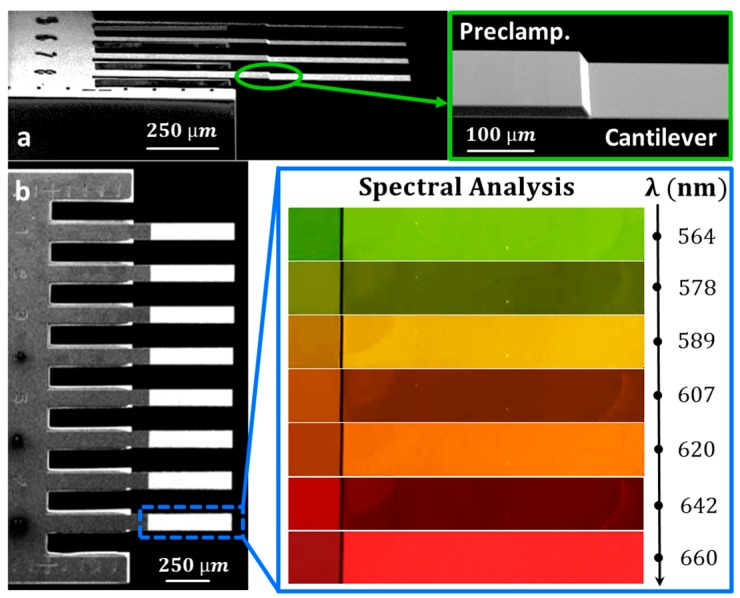
(**a**) SEM image in perspective view of four cantilevers; the top right inset (green box) is a zoomed SEM image near the cantilever clamping that evidences the frontier between the 6 µm thick pre-clamping structure fixed to the chip and the 1 µm thick cantilever; (**b**) SEM image of an entire array; the right inset (blue box) is a sequence of different bright-field images of a microcantilever obtained at different illumination wavelengths.

Commercial silicon cantilevers 500 µm long, 100 µm wide and 1 µm thick (CLA-500-010-08, Concentris GmbH, Basel, Switzerland) were chosen for the experimental characterization. The array of cantilevers is comprised of eight cantilevers connected to the chip through a 6 µm thick pre-clamping region, as shown in the scanning electron microscope (SEM) images of Figure 2a,b (inset green box). Bright spectral analysis, performed in the visible spectral range from 538 nm to 700 nm with steps of 1 nm, was achieved by using a 10X objective (LU Plan Fluor, Nikon, Tokyo, Japan), that ensures both a large detection area (≃1.2 mm × 1.5 mm) and a high spatial resolution of ~1 µm. In these experimental conditions, four cantilevers were measured simultaneously in just one minute. The wavelength dependent response of the SMMS apparatus was eliminated by normalizing the cantilever reflectivity with the reflectivity spectra of a bare silicon substrate. The following analysis will only refer to the normalized reflectivity R_norm_. In order to simplify the data analysis, reflectivity measurements have been performed by illuminating the sample surface at normal incidence angle. This condition was experimentally attained by shrinking the aperture of the adjustable diaphragm to reach a numerical aperture of the illumination light below 0.05. 

## 3. Results and Discussion

Bright-field images of a cantilever, with nominal thickness of 1 µm, and its pre-clamping, with nominal thickness of 6 µm, obtained at different wavelengths λ of the visible spectrum are shown in Figure 2b (blue box). The cantilever shows notable differences in reflectivity depending on the selected wavelength λ with multiple maxima (564 nm, 589 nm, 620 nm, 660 nm) and minima (578 nm, 607 nm, 642 nm) in the visible spectral range. This reflectivity modulation, known as Fabry-Perot interference, occurs because light can efficiently bounce multiple times between the opposite sides of a thin microcantilever [29,30,31]. At certain wavelengths, reflectivity is enhanced because these multiple reflections inside the microstructure generate constructive interference. Conversely, the reflectivity is suppressed for other wavelengths by destructive interference. At the pre-clamping region, this reflectivity modulation is negligible due to the absorption of silicon in the visible spectral range that avoids multiple reflections inside a thicker structure (6 μm). 

**Figure 3:**
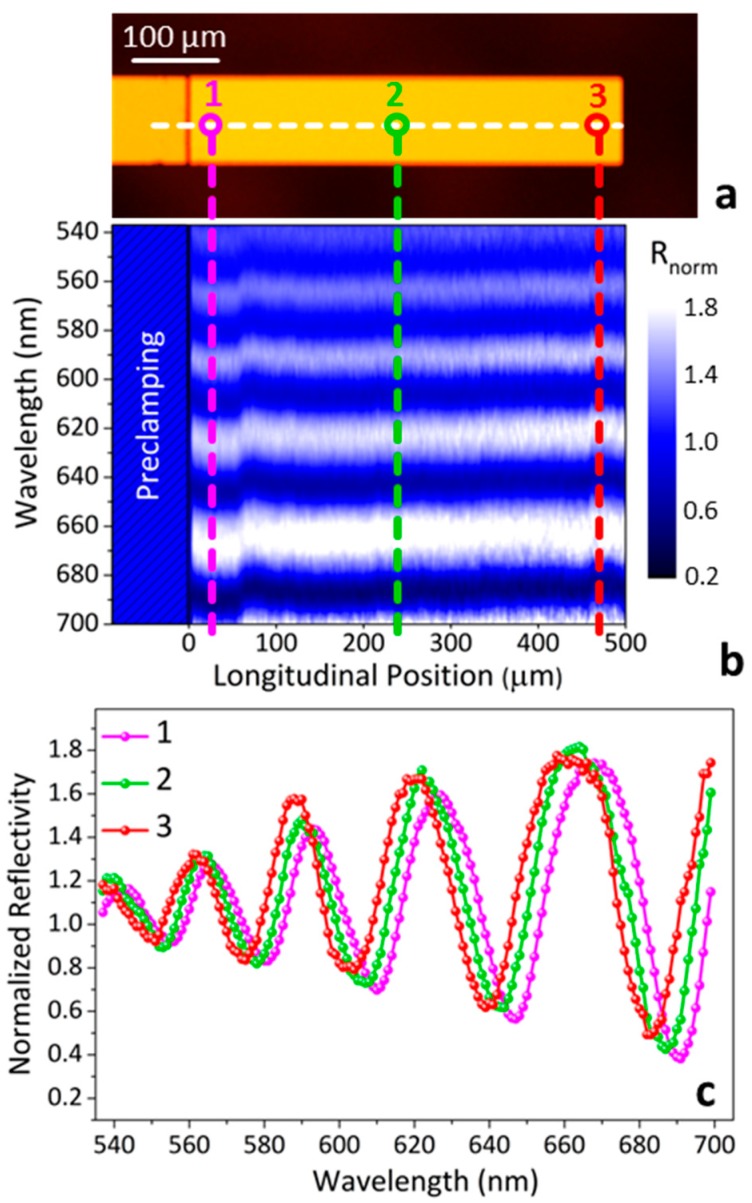
(**a**) Optical image of a cantilever; (**b**) color contour plot of the normalized reflectivity as a function of the light wavelength and of the longitudinal position along the cantilever (white dashed line in Figure 3a); (**c**) normalized reflectivity obtained at three different positions along the cantilever; near the clamped end (point 1, magenta), at the middle (point 2, green) and near the cantilever free end (point 3, red).

The color contour map of Figure 3b shows the wavelength-dependence of the normalized reflectivity measured along the cantilever longitudinal position (along the white dashed line drawn in Figure 3a). Each point measured along the cantilever exhibits a reflectivity modulation induced by Fabry–Perot interference. However, as highlighted by the vertical dashed lines of Figure 3b, the spectral positions of minima and maxima of reflectivity depend considerably on the position along the cantilever. This feature is more evident in Figure 3c, where the normalized reflectivity at three different cantilever positions is presented: near the clamping (point 1, magenta), at the middle (point 2, green) and close to the cantilever free end (point 3, red). The spectral shifts between the three reflectivity curves occur due to small cantilever thickness variations produced during the micro-fabrication process. 

### 3.1. Cantilever Thickness Mapping

The thickness d at each point on the cantilever surface was therefore calculated by fitting the experimental data with the following analytical expression [32]:

(1) $$R_{\text{norm}}=\left|{\left(\frac{\alpha_{+}}{\alpha_{-}}\right)}^{2}\right|\left|{\left(\frac{\alpha_{+}\alpha_{-}\left(e^{\frac{4i\pi dn_{c}}{\lambda }}-1\right)}{\alpha_{+}^{2}-\alpha_{-}^{2}e^{\frac{4i\pi dn_{c}}{\lambda }}}\right)}^{2}\right|$$

where $\alpha_{\pm }=\pm 1+n_{c}$, i is the imaginary unit and $n_{c}=n+i\kappa$ is the cantilever complex refractive index, where n is the refractive index and κ the extinction coefficient. Equation (1) is valid for a light beam with normal incidence angle, and it has been obtained by combining the Fresnel equations with the transfer matrix technique [32]. The cantilever thickness represents the only free parameter in the fitting, as the optical properties of the cantilever material are known. The dispersive behaviour of silicon and its optical properties have been considered according to Vuye *et al*. [33].

**Figure 4:**
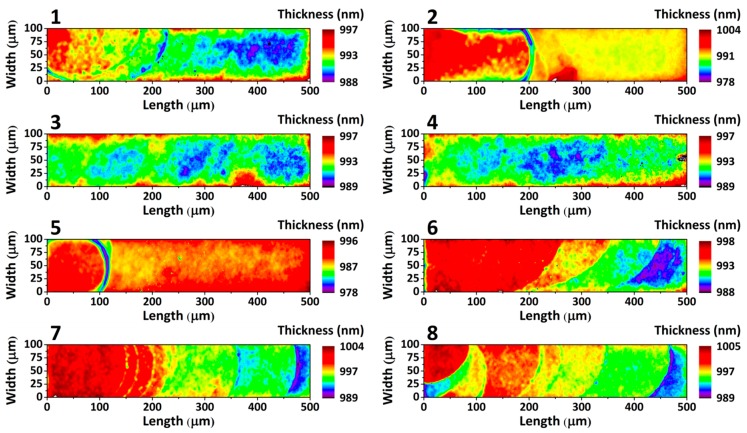
Thickness map of eight silicon cantilevers from the same chip array; the color bars represent the cantilever thickness along the microstructure in nanometers (nm).

The thickness mapping of an entire array of eight cantilevers is shown in Figure 4; the cantilever nominal thickness provided by the manufacturer is 1000 nm and the colour bar scales represent the measured cantilevers thickness in nanometers. The thickness mapping of each cantilever was obtained from around 50,000 non-linear fittings with a goodness of fit [34] always higher than 0.99. The SMMS technique is able to distinguish thickness variations of 0.1%; this accuracy is given by the spectral accuracy of our experimental setup and the goodness of the fitting procedure, and it does not depend neither on the thickness nor the material that we have to analyse. As in our experiments, we characterized a 1 μm thick layer; here, the absolute thickness accuracy is about 1 nm. Thickness maps reveal significant discrepancies between different cantilevers placed in the same array. For example, cantilevers 3 and 4 present good thickness homogeneity, with a root mean square (RMS) thickness variation of about 1 nm along all the microstructure. In turn, cantilevers 2 and 7 show higher thickness heterogeneity, with a RMS thickness variation of 3.5 nm. It is noteworthy that the fabrication process produces cantilevers that are thicker in the vicinity of the clamping region, getting thinner near the free end. 

Thickness characterization presented here was therefore confirmed also with SEM inspections by measuring the thickness on the edge of the cantilevers. Moreover, in analogy to the SMMS analysis presented above, SEM analysis also confirmed the presence of small structural defects on both top and bottom cantilever surfaces.

The high spatial resolution combined with the large detection area of the SMMS technique allows quick and precise inspection of micro-fabrication defects in the entire array of cantilevers. Remarkably, by using an optical objective with low numerical aperture, a 10 μm spatially-resolved spectral analysis of a full one-inch wafer can be performed in just two minutes.

The SMMS technique is a general method for the characterization of a large variety of thin films; the only requirement is that the material optical properties allow multiple reflections inside the thin film. Moreover, the sample surface roughness is not critical in the SMMS technique because scattered light due to surface roughness is still orders of magnitude lower than light reflected in the specular direction.

In a recent work, Salmon and co-workers [19] presented the thickness characterization of cantilevers using a standard micro-spectrophotometer. They reported a spatial resolution of 15 µm and a measurement time of 30 min for the characterization of one cantilever with the same planar dimensions as the ones used in this work. The spatial variation in thickness mapped by the SMMS technique presented here provides higher spatial resolution (1 µm), and the measurement of four cantilevers took only one minute. Moreover, although micro-fabrication defects can be easily observed in a routine inspection with a scanning electron microscope, even a qualitative inspection is time-consuming, in some cases destructive, and a quantitative analysis of microstructures is generally complex to perform [19,35].

### 3.2. Effect of Thickness Variability on Microcantilever Mechanical Properties

As seen from Figure 4, the thickness of suspended micromechanical devices can show inhomogeneities, even for devices fabricated very close on the same array. The defects could be due to the etching steps, as they are more pronounced close to the clamping regions. As most of the mechanical parameters of a resonator strongly depend on its thickness, it represents the most important geometrical factor in mechanical variability. The error produced by thickness inhomogeneity can play a relevant role in a large variety of applications, such as calibration of cantilever spring constant in single molecule force-spectroscopy and scanning-probe microscopy or mass and surface stress sensitivities in cantilever sensing applications [25,36,37,38]. A common strategy in nanomechanical sensors is the use of a reference cantilever placed in the same array to eliminate common noise and drift signals from non-specific interactions, variations in temperature, analyte medium refractive index, pressure, *etc*. [39,40,41]. This approach assumes that the reference and sensor cantilevers have the same mechanical responsivity. Although the effect of the thickness inhomogeneity has been commonly disregarded in literature, it can produce appreciable mechanical variability between cantilevers placed in the same array, and, for this reason, thickness variability needs to be considered to avoid misinterpretation of results. 

The accurate thickness mapping performed with the SMMS technique allows for calculating the variations in mechanical responsivity produced by the thickness inhomogeneity; the mechanical responsivity can be easily observed with a nanomechanical detection setup, as the beam deflection method, that is able to characterize the dynamical properties of a vibrating mechanical structure [42,43,44,45]. For each cantilever shown in Figure 4, we analyse: (i) the *mass sensitivity* $S_{\text{mass}}=\Delta f/\Delta m$, defined as the cantilever frequency shift Δf per unit of mass Δm added onto the cantilever and and (ii) the *surface stress sensitivity* $S_{\text{stress}}=\Delta z/\Delta \sigma$, defined as the displacement of the cantilever free end Δz per unit of uniform and isotropic differential surface stress Δσ exerted on the cantilever surface. In order to take into account the cantilever thickness irregularities on both mass and surface stress sensitivities, numerical analysis is needed. 

**Figure 5:**
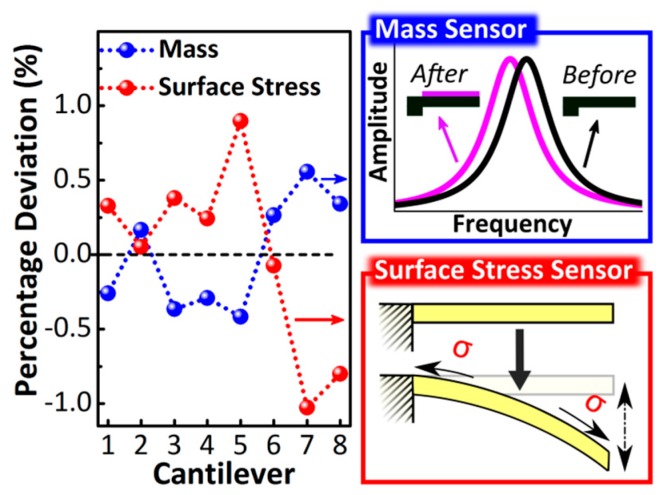
Percentage deviation of the mass and surface-stress sensitivities with respect to their average values for each of the eight measured cantilevers. (Top right blue box) Schematics of the effect of the mass loading on the resonance frequency of a cantilever; the resulting downshift of the resonance frequency is proportional to the added mass. Mass sensitivity was evaluated by calculating the resonance frequency shift produced by the addition of a uniform mass layer deposited on the top cantilever surface. (Bottom right red box) Schematics of the effect of the surface stress on the out-of-plane displacement of a cantilever; the cantilever displacement is proportional to the surface stress applied. Surface stress sensitivity was evaluated by calculating the displacement of the free cantilever end induced by a uniform and isotropic surface stress exerted on the upper cantilever surface.

Finite element analysis, performed on cantilevers with the thickness inhomogeneity shown in Figure 4, gives an average mass and surface stress sensitivity of 24 Hz/ng and 3.2 nm/(mN/m), respectively; more technical details about the numerical analysis can be found in Appendix B. The percentage deviation of the cantilever mass and surface stress sensitivities compared to their average values are shown in Figure 5 for each cantilever of the array under study; numerical calculations show a deviation in mass and surface–stress sensitivity between cantilevers within the same array of up to 1% and 2%, respectively.

The differences in the mechanical properties between cantilevers of the same array will be examined in a hypothetical sensing experiment: the deformation induced by the immobilization of a densely packed single-strand DNA layer exerting 150 mN/m of surface stress on the top cantilever surface. The deflection differences between cantilevers of the same array can reach up to 10 nm. This difference in sensitivity must be taken into account when comparing the responses between the reference and sensor cantilevers, because its magnitude is of the same order of other noise sources commonly taken into account in a biosensing experiment [26]. For example, the absorption of a DNA monolayer on similar microcantilevers coated with the complementary strand produces a bending only 6 nm larger than that on microcantilevers carrying the negative control sequence [42]. Up to now, the follow up of these small signals has demanded the prior calibration of the microcantilevers by application of heat pulses to choose microcantilevers of similar mechanical responsivity as reference [43]. The experimental characterization presented here will allow the calibration of the devices immediately after the fabrication process, so the expected responsivity could be predicted and the cited experimental calibration avoided.

Furthermore, assessing the mechanical variability becomes even more important when using large cantilever arrays [44] and in statistical analysis of measurements for cantilever biosensing [45]. Depending on the fabrication process and mainly on the quality of the silicon on insulator (SOI) wafers used for the cantilever fabrication, the thickness inhomogeneity can be much higher and the differences in responsivity between cantilevers from different arrays or from different wafers can increase dramatically [19].

## 4. Conclusions

In this work, we demonstrated the fast thickness characterization of thin suspended films by using a novel optical technique, called Spatially Multiplexed Micro-Spectrophotometry (SMMS) in bright field mode. The great advantage compared to standard micro-spectrophotometry is that the spectral analysis of a large sample area is performed in a parallel way, thus ensuring at least two orders of magnitude shorter analysis time than state-of-the-art micro-spectrophotometers. We demonstrate the capability of the SMMS technique by mapping, in just two minutes, the thickness of an array of commercial cantilevers with micrometrical spatial resolution. The present technique, able to map the cantilever thickness with 1 nm vertical accuracy, allows the rapid inspection of tiny structural defects over the whole device, making it possible to predict the mechanical responsivity of the devices and to assess deviations produced during the nanofabrication process. The fast and high-resolution spatially-resolved analysis of large sample areas is now feasible, offering a new experimental tool for a multitude of industrial and scientific applications of nanotechnology. 

## Appendix A: Raw Data Normalization

Raw data need to be normalized with a reference sample for two reasons: (i) the illumination source, the CCD camera and all the optical components of the experimental setup are wavelength-dependent and (ii) the light source does not illuminate the whole sample surface with spatial homogeneity. The practical procedure for the normalization is obtained by dividing pixel by pixel each spectral component λ of the sample reflectivity R(x,y,λ) by the reflectivity $R_{\text{ref}}\left(x,y,\lambda \right)$ of a reference material, *i.e.*,

(A.1) $$R_{\text{norm}}\left(x,y,\lambda \right)=\frac{R\left(x,y,\lambda \right)}{R_{\text{ref}}\left(x,y,\lambda \right)},$$

where *x* and *y* are the spatial coordinates of the sample surface.

## Appendix B: Finite Element Analysis of Cantilevers with Thickness Inhomogeneity.

In order to take into account the cantilever thickness irregularities on mass and surface stress sensitivities, finite element simulations with the commercial software Comsol^®^ (Stockholm, Sweden) were performed. The experimental cantilever thickness is imported in the numerical simulations by generating a parametric surface from the experimental thickness mappings of Figure 4. The top cantilever surface is given by this parametrical surface, while the bottom surface is completely flat.

Mass sensitivity was calculated by evaluating the resonance frequency shift of the first flexural mode before and after the addition of a uniform mass layer onto the cantilever. Numerical simulations after mass deposition were obtained by adding an infinitely thin layer of 10^−5^ kg/m^2^ on the bottom face of each of the eight cantilevers.

Surface stress sensitivity was evaluated by calculating the deflection of the cantilever free end subjected to an isotropic and uniform surface stress onto one side of the cantilever surface. In this case, a shell of 10 nm on the bottom face of each cantilever was added and an initial surface stress of 0.08 N/m was considered. Numerical simulations were obtained by considering a Young modulus E = 170 GPa, a material density of 𝜌 = 2329 kg/ m^3^ and a Poisson’s ratio of 𝜈 = 0.28.
